# Multi-omic alterations of the SWI/SNF complex define a clinical subgroup in lung adenocarcinoma

**DOI:** 10.1186/s13148-022-01261-3

**Published:** 2022-03-17

**Authors:** Paola Peinado, Alvaro Andrades, Marta Cuadros, Maria Isabel Rodriguez, Isabel F. Coira, Daniel J. Garcia, Maria S. Benitez-Cantos, Carlos Cano, Eduardo Zarzuela, Javier Muñoz, Claudia Loidi, Monica Saiz, Pedro P. Medina

**Affiliations:** 1 Department of Biochemistry and Molecular Biology I, University of Granada, Campus Fuentenueva s/n, 18071 Granada, Spain; 2 GENYO, Centre for Genomics and Oncological Research: Pfizer/University of Granada/Andalusian Regional Government, Granada, Spain; 3 Health Research Institute of Granada (Ibs.Granada), Granada, Spain; 4 Department of Biochemistry and Molecular Biology III and Immunology, University of Granada, Granada, Spain; 5 School of Pharmaceutical Sciences, University of Geneva, Geneva, Switzerland; 6 Institute of Pharmaceutical Sciences of Western Switzerland, University of Geneva, Geneva, Switzerland; 7 Department of Computer Science and Artificial Intelligence, University of Granada, Granada, Spain; 8 Proteomics Unit, Spanish National Cancer Research Center, CNIO. Proteored-ISCIII, Madrid, Spain; 9 Pathological Anatomy, Universitary Hospital Cruces, University of Pais Vasco, Gipuzkoa, Spain

**Keywords:** Multi-omics, Lung cancer, Lung adenocarcinoma, SWI/SNF complex, Epigenetics, Prognosis

## Abstract

SWI/SNF complexes are major targets of mutations in cancer. Here, we combined multiple “-omics” methods to assess SWI/SNF composition and aberrations in LUAD. Mutations in lung SWI/SNF subunits were highly recurrent in our LUAD cohort (41.4%), and over 70% of the mutations were predicted to have functional impact. Furthermore, SWI/SNF expression in LUAD suffered an overall repression that could not be explained exclusively by genetic alterations. Finally, SWI/SNF mutations were associated with poorer overall survival in TCGA-LUAD. We propose SWI/SNF-mutant LUAD as a separate clinical subgroup with practical implications.

**Supplementary Information:**

The online version contains supplementary material available at 10.1186/s13148-022-01261-3.

## Introduction

Lung adenocarcinoma (LUAD) is the main histological subtype of lung cancer, which is currently the deadliest cancer worldwide [1]. The poor outcome of LUAD patients may be improved by an early diagnosis and a personalized clinical approach, both of which can be facilitated by next-generation sequencing (NGS).

NGS studies have identified that the multiprotein complex SWI/SNF (*SWitch/Sucrose Non-Fermentable*) is mutated in almost 25% of human neoplasias [2, 3]. Before the era of NGS, our group discovered that *SMARCA4* is frequently inactivated by truncating mutations in LUAD [4–6]. Together with *SMARCA4*, other SWI/SNF subunits, such as *ARID1A*, are recurrently mutated in LUAD and considered as LUAD driver genes [7]. Recently, our group has shown that more than 76% of LUAD cell lines have at least one mutated SWI/SNF subunit [6]. However, the exact composition of the SWI/SNF complex in LUAD is currently unknown and we lack an integration of the genetic and transcriptional profile of this complex in order to facilitate a practical transfer to the clinic.

For all these reasons, we aimed to identify the proteins that form the SWI/SNF complexes in lung epithelial cells as well as their molecular alterations in LUAD primary tumors, and more importantly, the clinical application of this in-depth study.

## Methods

### Characteristics of lung adenocarcinoma patients

DNA and RNA from 70 lung adenocarcinoma (LUAD) tumors and their paired normal adjacent tissues were obtained from the Basque Biobank (www.biobancovasco.org) and were processed following standard operating procedures. Lung adenocarcinoma patients were diagnosed from August 2008 to January 2016. The main characteristics of these 70 patients are shown in Additional file 2: Table S1. More information about this patient cohort is detailed in Additional file 1: Supplementary Methods.

### Cell culture

Normal bronchial epithelial cells, NL20, were grown under standard culture conditions (37ºC, 5% carbon dioxide) in Ham’s F12 medium with 4%FBS, 2.0 mM L-glutamine, 1.5 g/L sodium bicarbonate, 2.7 g/L glucose, 0.1 mM nonessential amino acids, 1 μg/mL transferrin, 5 μg/mL insulin, 10 ng/ml EGF, and 500 ng/mL hydrocortisone.

### Gene capture and targeted sequencing

The baits for the gene capture were designed using the NimbleDesign software (Roche, v4.0). The baits were targeted against 20 SWI/SNF genes and the top 10 LUAD drivers identified by Bailey and colleagues [7] *(*Additional file 2: Table S2). We included the known LUAD drivers as positive controls (see Additional file 1: Supplementary Methods).

### Deep sequencing data analysis

We aligned the raw reads to the hg38 human genome using BWA-MEM. Details on the pipelines, software versions and external data sources are discussed in Additional file 1: Supplementary Methods.

### Real-time quantitative polymerase chain reaction

Real-time quantitative PCR (RT-qPCR) was optimized using the Applied Biosystems 7900HT Real-Time PCR System with cDNA prepared after a reverse transcription of 1 μg total RNA (RevertAid RT Kit, Thermo Scientific). All qPCR reactions followed the KAPA SYBR® FAST qPCR Master Mix recommendations. Relative expression was calculated using *GAPDH* as housekeeping gene and applying the DDCt method. Primers for each gene are shown in Additional file 2: Table S3. All experiments were carried out in duplicate or triplicate.

### Immunoprecipitation

5 mg of protein from NL20 lysates was immunoprecipitated following the conditions detailed in Additional file 1: Supplementary Methods.

### Mass spectrometry

LC–MS/MS was done by coupling an UltiMate 3000 HPLC system to a Q Exactive Plus mass spectrometer (Thermo Fisher Scientific) (see Additional file 1: Supplementary Methods).

### Mass spectrometry data analysis

Raw files were processed with MaxQuant (v 1.6.2.6a) using the standard settings against a human protein database (UniProtKB/Swiss-Prot, 20,373 sequences) supplemented with contaminants (more information in Additional file 1: Supplementary Methods).

### In silico analysis of the SWI/SNF complex in lung adenocarcinoma patients

We downloaded mutation, gene expression, and clinical data of LUAD patients from The Cancer Genome Atlas (TCGA-LUAD project, last updated October 1, 2019). Analyses are detailed in Additional file 1: Supplementary Methods.

### Statistical analyses

Unless otherwise specified, all statistical analyses were performed using R (version 3.6.1). Normality of the data was assessed using quantile–quantile plots and data transformations and statistical tests were chosen accordingly. For more details about the statistical analyses, see Additional file 1: Supplementary Methods.

## Results

Although tissue specificity is a widely known trait of the SWI/SNF complex, no studies have analyzed SWI/SNF composition in a lung epithelial cell model [8]. For this reason, first we aimed to identify which subunits constitute the SWI/SNF complex in lung epithelial cells. We performed an endogenous immunoprecipitation of SMARCA4 followed by liquid chromatography-tandem mass spectrometry/mass spectrometry (LC–MS/MS) in NL20, a non-tumorigenic bronchial epithelial cell line. Twenty SWI/SNF subunits were pulled down along with SMARCA4 (Additional file 1: Fig. S1, Additional file 2: Tables S4–S5). From now on, we will refer to the SWI/SNF subunits that were identified in the immunoprecipitation, plus SMARCA4 and SMARCA2, as “lung SWI/SNF subunits.”

**Fig. 1:**
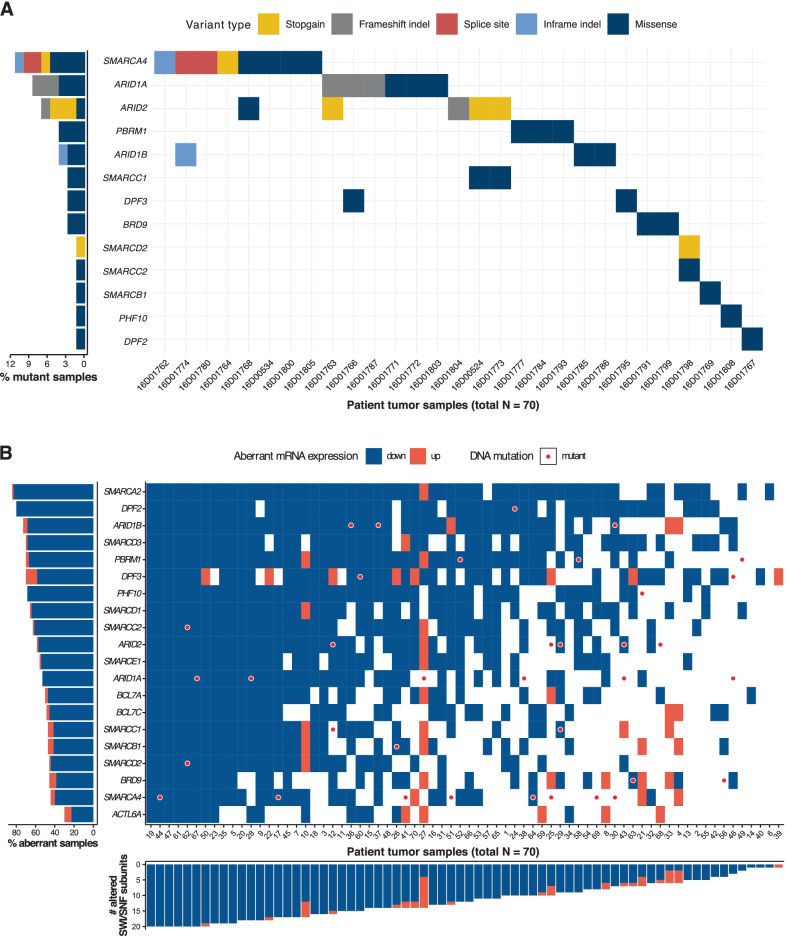
Mutational and transcriptional study of SWI/SNF in LUAD primary tumors (**A**) Mutation profile of the 20 lung SWI/SNF complex subunits in our LUAD cohort. Y axis represents all the subunits that had at least one genetic alteration in at least one LUAD patient. X axis gathers all LUAD patients with a mutant SWI/SNF complex. On the left, mutation frequencies of these lung SWI/SNF subunits in our LUAD patients. (**B**) Tile plot of the mRNA expression of the lung SWI/SNF subunits in our LUAD cohort. Blue colors correspond to those genes that showed ≤ -2 × expression in the tumor sample than in the matched normal sample. Orange colors are displayed when a gene was expressed ≥ 2 × in the tumor. White colors correspond to those expression values that did not reach the thresholds that we defined for upregulation or downregulation. Red circles are present when a certain gene was mutated in a specific patient. On the left side, lung SWI/SNF genes are arranged based on downregulation percentage in our LUAD patients. At the bottom of the tile plot, our 70 LUAD patients are arranged based on the number of lung SWI/SNF subunits that were downregulated in their tumors

To examine the mutational status of the lung SWI/SNF subunits, we performed targeted DNA sequencing in seventy LUAD primary tumors and twenty-seven of the matched normal adjacent samples. We used the information from a paired analysis on the twenty-seven matched tumor-normal pairs to optimize a pipeline for unpaired mutation calling in the seventy primary LUAD tumors (Additional file 1: Supplementary Methods and Supplementary Note). We analyzed the twenty lung SWI/SNF subunits that had good quality sequencing. In these lung SWI/SNF subunits, we found 38 point mutations and small indels in our LUAD patient cohort (*N* = 70). Twenty-nine (41.4%) of the primary tumors harbored at least one mutation in a lung SWI/SNF subunit (Fig. 1A). *SMARCA4* was the most commonly mutated SWI/SNF gene (11.4% of samples), followed by *ARID1A* (8.6%), *ARID2* (7.1%), *ARID1B* (4.3%), and *PBRM1* (4.3%).

Next, to investigate the mutation frequencies in external LUAD cohorts, we examined publicly available data from TCGA-LUAD (last updated on October 1, 2019. *N* = 567). The distributions of clinical parameters were comparable between the two cohorts (Additional file 2: Table S1). Our cohort showed similar but slightly higher mutation frequencies in the lung SWI/SNF genes (Additional file 1: Fig. S2A). Overall, the total mutation frequency of the SWI/SNF complex was 41.4% in our cohort and 30.0% in TCGA-LUAD, possibly due to a greater coverage in our protocol or to differences in data analysis protocols. Furthermore, regardless of the cohort, *SMARCA4*, *ARID1A*, and *ARID2* were the SWI/SNF subunits that accumulated the highest number of truncating mutations.

To predict the functional impact of missense mutations, we used the SIFT algorithm [9] (Additional file 1: Supplementary Methods and Fig. S2B). Based on SIFT predictions, more than half of the missense mutations in our cohort (64%, 16/25) and in the external data (65%, 103/159) were “deleterious.” Overall, considering the truncating mutations and the predicted deleterious missense mutations, more than 70% of the SWI/SNF mutations may have a functional impact.

To complement our mutational study, we analyzed the mRNA levels of the lung SWI/SNF subunits in our cohort. To measure expression accurately, we used RT-qPCR. We found that all lung SWI/SNF subunits were significantly downregulated in LUAD primary tumors compared to their matched normal adjacent samples (FDR-adjusted *p* < 0.05, Additional file 1: Fig. S3). We set a fold change threshold of + 2/− 2 between the tumor and the paired normal sample to consider a subunit to be up- or downregulated, respectively. Remarkably, most lung SWI/SNF subunits consistently showed lower expression in most tumors when compared to their paired normal tissues (Fig. 1B, Additional file 1: Fig. S3). We found 42 tumors (60%) that had more than 10 downregulated subunits. On average, each lung SWI/SNF subunit was downregulated in 57% of LUAD patients. The top downregulated SWI/SNF subunit was *SMARCA2* (82% of the cases). Similar results have been observed in other tumors where *SMARCA2* was found to be epigenetically repressed [10–12]. Moreover, none of the top 5 downregulated subunits (*SMARCA2*, *DPF2*, *SMARCD3*, *PHF10* and *SMARCD1*) were among the top 5 most frequently mutated subunits. More generally, only 5/11 (45.5%) truncating mutations and 13/23 (56.5%) missense mutations were associated with more than a twofold decrease in expression. Overall, these findings suggest a profound silencing in the expression of the whole SWI/SNF machinery in LUAD and that genetic alterations are not the only cause of SWI/SNF inactivation.

**Fig. 2:**
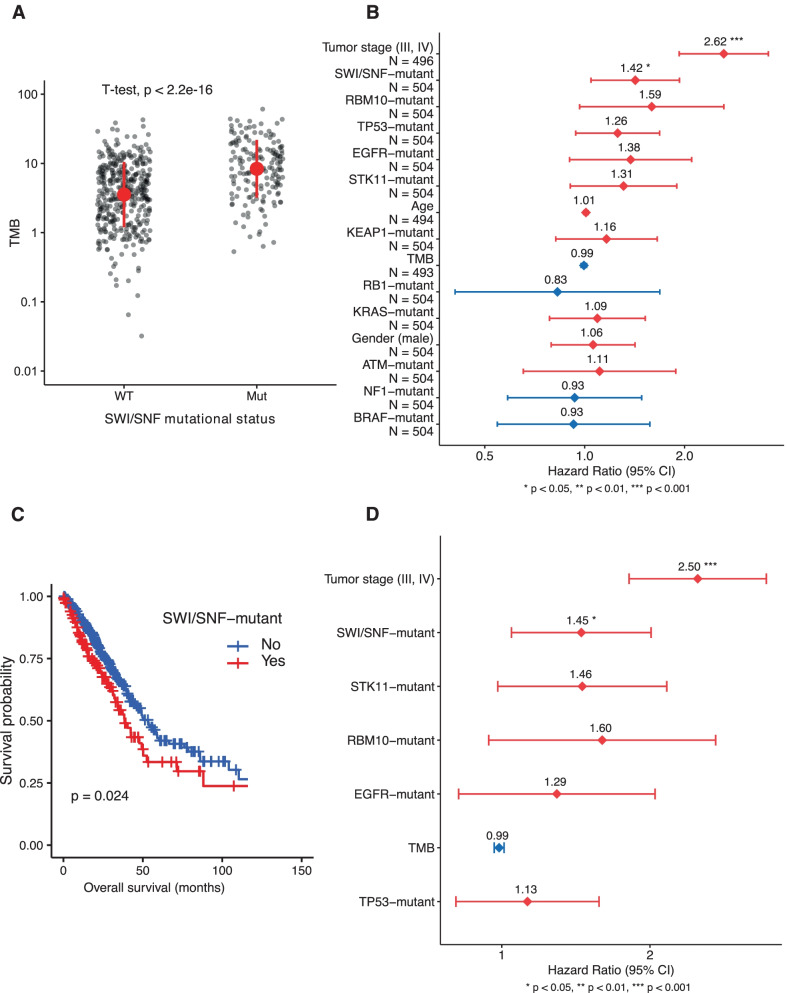
Clinical analyses with the mutational status of the lung SWI/SNF complex (**A**) Tumor mutation burden (TMB), defined as the number of non-silent mutations per Mb as estimated by Hoadley et al. [21], in SWI/SNF wild type vs SWI/SNF mutant patients in TCGA-LUAD. The red dot and lines represent the mean and standard deviation of the log_10_(TMB) values, respectively. A two-tailed Student's *t* test was performed on the log_10_(TMB) values. (**B**) Univariate Cox Proportional-Hazards regression on mutation and clinical covariates from TCGA-LUAD. All variables included in the model are sorted by statistical significance (*p*-value). (**C**) Kaplan–Meier curves grouping the TCGA-LUAD cohort by the mutational status of SWI/SNF complex (Logrank test). (**D**) Multivariate Cox Proportional-Hazards regression on mutation and clinical TCGA-LUAD covariates. All variables included are sorted by statistical significance (*p*-value)

In our mutational analysis, we also observed that SWI/SNF-mutant tumors from TCGA-LUAD showed a significantly higher Tumor Mutation Burden (TMB) than SWI/SNF-wild type tumors (*p* < 0.05) (Fig. 2A). Furthermore, we evaluated whether the mutational status of the lung SWI/SNF subunits was associated with LUAD overall survival in the TCGA-LUAD cohort. To select variables for a multivariate Cox analysis, we first performed univariate Cox analyses on each of the variables under study and we selected those with *p* < 0.2. We considered mutations in SWI/SNF and LUAD driver genes, TMB, and other clinically relevant covariates (Additional file 1: Supplementary Methods). In the univariate analysis, none of the individual SWI/SNF subunits were significantly associated with overall survival (OS), but SWI/SNF mutations altogether were significantly associated with poorer OS (HR = 1.42; 95% CI: 1.04–1.93; *p* = 2.5·10–2) (Fig. 2B and C). These observations led us to consider the SWI/SNF complex as a single functional unit. Mutations in none of the top 10 LUAD driver genes from Bailey et al. [7] were significantly associated with OS (Additional file 1: Fig. S4A–J). Next, all variables with *p* < 0.2 in the univariate analysis were used for a multivariate analysis. According to this analysis, the SWI/SNF mutational status is an independent prognostic factor associated to shorter OS in LUAD patients (HR = 1.45; 95% CI: 1.05—2.01; *p* = 2.56·10^–2^) (Fig. 2D). Therefore, the lung SWI/SNF mutational status distinguishes between two clinically different subgroups.

## Discussion

In this work, we have analyzed the status of the SWI/SNF complex in LUAD by combining multiple “-omics” approaches. The distribution of SWI/SNF mutations in our cohort was comparable to that of TCGA-LUAD, and over 70% of the identified mutations were predicted to have functional impact. These results support the key role of SWI/SNF in cancer [2, 3, 13].

To date, although downregulation events have previously been described in certain SWI/SNF subunits [14, 15], our study is the first one that reveals a general downregulation of the whole SWI/SNF complex in LUAD. The functional implications that this observation could have in tumorigenesis are supported by many years of study of the SWI/SNF complex in several tumor types and biological contexts (reviewed in [2]).

Interestingly, we also observed that SWI/SNF-mutant tumors had a higher TMB than SWI/SNF-wild type tumors, supporting the function of the SWI/SNF complex in maintenance of genome integrity [16, 17]. Moreover, this observation highlights the potential use of the mutational status of the SWI/SNF complex as a biomarker of response to immune checkpoint inhibitors, which benefits from elevated tumor mutation burdens [18–20]. Importantly, we have shown for the first time that, when evaluated as a whole, SWI/SNF complex mutations correlate with poor prognosis in LUAD. On the other hand, mutations in well-known biomarkers such as *EGFR* and *KRAS* were not significantly associated with overall survival in the analyzed cohort. This reinforces the clinical relevance of analyzing SWI/SNF mutations in LUAD alongside other established prognostic factors. Indeed, the mutational status of the lung SWI/SNF complex was an independent prognostic factor when evaluated alongside the TMB and other clinical variables commonly associated with survival.

Overall, we propose the lung SWI/SNF as a functional unit whose recurrent mutations predict a worse clinical outcome. Moreover, there is a major downregulation of the SWI/SNF complex in LUAD that can only be partly attributed to mutations. Taken together, our findings highlight a major role of genetic and epigenetic alterations in the SWI/SNF complex in LUAD that can have clinical applications.

## Supplementary Information

**Additional file 1**. Supplementary Methods, Supplementary Note and Supplementary Figures S1–S4.

**Additional file 2**. Supplementary Tables S1–S6.

## Data Availability

Human DNA sequencing data have been uploaded to the European Genome-phenome Archive (EGA) under the accession EGAD00001005930. The mass spectrometry proteomics data have been deposited to the ProteomeXchange Consortium via the PRIDE partner repository with the dataset identifier PXD017397. The results published here are in part based upon data generated by The Cancer Genome Atlas (TCGA) managed by the NCI and NHGRI. Information about TCGA can be found at http://cancergenome.nih.gov. TCGA-LUAD can be accessed at the Genomic Data Commons Data Portal (https://portal.gdc.cancer.gov/).

## Abbreviations

FDR: False Discovery Rate
LC–MS/MS: Liquid Chromatography-tandem Mass Spectrometry/Mass Spectrometry
LUAD: Lung adenocarcinoma
IP: Immunoprecipitation
RT-qPCR: Real-time quantitative polymerase chain reaction
SWI/SNF: SWitch/Sucrose Non-Fermentable
TCGA: The Cancer Genome Atlas
TMB: Tumor mutation burden
