# Supplementary material for: Multi-omic alterations of the SWI/SNF complex define a clinical subgroup in lung adenocarcinoma
Source: Clin Epigenetics. 2022 Mar 17;14:42. doi: 10.1186/s13148-022-01261-3 (PMC8931969; doi:10.1186/s13148-022-01261-3)

# Supplemental Information

Table of contents


1) **Supplemental Methods**
    1.1. Characteristics of lung adenocarcinoma patients
    1.2. Cell culture
    1.3. Gene capture and targeted sequencing
    1.4. Deep sequencing data analysis
    1.5. Evaluation of the functional impact of missense mutations
    1.6. Immunoprecipitation
    1.7. Sample preparation for proteomic analysis
    1.8. Mass spectrometry
    1.9. Mass spectrometry data analysis
    1.10. In silico analysis of the SWI/SNF complex in lung adenocarcinoma patients
    1.11. Real-time quantitative polymerase chain reaction
    1.12. Statistical analysis
2) **Supplemental Note**
    2.1. Performance of different mutational analysis approaches
3) **Supplemental References**
4) **Supplemental Figure Legends**
5) **Supplemental Figures**
6) **Supplemental Tables**

## 1) Supplemental Methods:

**Characteristics of lung adenocarcinoma patients**

DNA and RNA from 70 lung adenocarcinoma (LUAD) tumors and their paired normal adjacent tissues were obtained from the Basque Biobank (www.biobancovasco.org) and were processed following standard operating procedures. Lung adenocarcinoma patients were diagnosed from August 2008 to January 2016. The main characteristics of these 70 patients are shown in ***Supplementary Table S1.***

The inclusion criteria for our patients required the following information: 1) histological diagnosis of lung adenocarcinoma, 2) availability of demographic and clinical data, 3) availability of DNA and RNA samples for genomic and transcriptomic analyses, and 4) provision of signed informed consent.

An independent experienced pathologist confirmed all diagnoses with pathological examinations.

Our patient cohort was homogeneous and no statistically significant differences were found in terms of age, sex, stage, relapse, or survival when comparing the different subgroups. Patients included 50 men and 20 women, whose ages ranged from 47.6 to 83.2 years. This cohort had a median age of 66.1 years at the diagnosis of LUAD, a median time to relapse of 17.4 months, and an overall survival of 20.1 months.

**Cell culture**

Normal bronchial epithelial cells, NL20, were grown under standard culture conditions (37ºC, 5% carbon dioxide) in Ham's F12 medium with 4%FBS, 2.0 mM L-glutamine, 1.5 g/L sodium bicarbonate, 2.7 g/L glucose, 0.1 mM nonessential

amino acids, 1 µg/mL transferrin, 5 µg/mL insulin, 10 ng/ml EGF, and 500 ng/mL hydrocortisone.

**Gene capture and targeted sequencing**

The baits for the gene capture were designed using the NimbleDesign software (Roche, v4.0). The baits were targeted against 20 SWI/SNF genes and the top 10 LUAD drivers identified by Bailey and colleagues(8) (***Supplementary Table S2***). We included the known LUAD drivers as positive controls.

The library preparation and gene capture protocol was performed using the SeqCap EZ Choice Enrichment kit. The design spanned the exons (including UTRs) of all target genes. Each target was padded by 10 nucleotides at 5' and 3' in order to ensure the inclusion of splice regions.

300 ng of genomic DNA were fragmented using a Covaris S2 sonicator yielding 180-220 bp fragments. After end repair and adapter ligation, the adapter-ligated fragments were amplified by PCR (9 cycles). The PCR fragments were purified and the fragments with the correct size were selected. DNA was denatured and hybridized against biotinylated probes, which were then captured using streptavidin-bound magnetic beads. The DNA bound to the beads was isolated and amplified by PCR (14 cycles). The quality and the concentration of the DNA was evaluated using NanoDrop (Thermo Scientific) and BioAnalyzer (Agilent). The paired-end sequencing was performed on a NextSeq 500 instrument (Illumina) using a NextSeq 500/550 Mid Output Kit (Illumina), 2x150 cycles.

**Deep sequencing data analysis**

The quality of the raw FASTQ sequencing files was evaluated using FastQC (v0.11.5, http://www.bioinformatics.babraham.ac.uk/projects/fastqc). Then, the adapter sequences were removed using Cutadapt [1] with the following options: -b AGATCGGAAGAGC -B AGATCGGAAGAGC –q 20 –m 50.

After trimming the adapters, the reads were aligned to the hg38 human genome (http://hgdownload.cse.ucsc.edu/goldenPath/hg38/bigZips) using BWA-MEM (v0.7.13-r1126) with the -M option. Afterwards, we used Picard (v2.1.1) to convert the SAM files to BAM format, sort the BAM files and mark PCR duplicates. Quality metrics were collected using Qualimap (v2.2.1) [2] and MultiQC (v1.7) [3].

For the paired variant calling on the 27 tumor-normal matched samples, we used Mutect2 (GATK version 4.1.4.0). We generated a panel of normals (PoN) using the sequencing data from our 27 sequenced normal samples, including any mutation found in at least one normal sample (`--min-sample-count 1` option in `CreateSomaticPanelOfNormals`). Although using related normal samples for the creation of the PoN is known to introduce slight biases in the results, we concluded that it was a better approach than using an external PoN because our PoN was able to better capture the sequencing artifacts that were specific to our experimental approach. We ran Mutect2 in paired mode using default parameters and providing our PoN and an external germline resource from gnomAD v3 (https://storage.googleapis.com/gnomad-public/release/3.0/vcf/genomes/gnomad.genomes.r3.0.sites.vcf.bgz). We then used `FilterMutectCalls` with default parameters to filter out false positive variants. We merged, normalized and left-aligned the mutations that passed the filters using bcftools (HTSlib version 1.7) and we annotated the multi-sample

VCFs using ANNOVAR (version 2017-07-17) with the following databases: ensGene (v20170912), 1000g2015aug_all, exac03, avsnp150 and dbnsfp33a. Variants with a minor allele frequency ≥ 0.01 in 1000 Genomes or ExAc were excluded. We also excluded synonymous mutations and non-coding mutations.

For the non-paired mutational analysis, we combined two approaches. In our first approach, we used BCFtools applying the following filters: individual variant QUAL ≥ 20 and either total coverage ≥ 8 or ≥ 5 mutant reads. We also flagged as ‘LowFreq’ the mutations that had a mutant allele frequency below 20%:

```
bcftools mpileup -f hg38.fa -R primary_targets.bed -q 1 -Q 13 -a ‘FORMAT/AD’ -Ou ${bam} | bcftools call -vmO z | bcftools filter -e “%QUAL<20 | ((FMT/AD[0:0]+FMT/AD[0:1])<8 & FMT/AD[0:1]<5)” -s “LowQual” -O u | bcftools filter -e “FMT/AD[0:1]/(FMT/AD[0:0]+FMT/AD[0:1])<0.2” -s “LowFreq” -m + -O u | bcftools sort -O z > ${out}.vcf.gz
```

We merged and annotated the resulting VCFs as described above. We then filtered out mutations present in any of our 27 normal samples, mutations present in ExAc or 1000 Genomes Project at frequencies above 0.01, mutations that overlapped simple repeats or low complexity regions according to RepeatMasker (downloaded from http://hgdownload.cse.ucsc.edu/goldenpath/hg38/database/rmsk.txt.gz), and synonymous or non-coding mutations.

In our second approach, we applied Mutect2 in non-paired mode using default parameters, our in-house PoN and gnomAD v3 as a germline resource. We compared our two approaches for the non-paired analyses and we individually

evaluated the discrepancies between the two pipelines using Integrative Genomics Viewer (v2.3.94) and public databases (see ***Supplementary Note***). After our thorough evaluation, we decided to combine the results from the BCFtools analysis with manually 'rescued' mutations from the Mutect2 approach after careful inspection of the Mutect2-exclusive mutations. We flagged such mutations as 'Mutect2'.

**Evaluation of the functional impact of missense mutations**

To predict the functional impact of missense mutations, SIFT was used (integrated in Ensembl VEP 95) for both our mutational data and TCGA-LUAD mutations. Only one consequence per variant allele was kept. We considered 'possibly damaging' mutations as 'damaging', and 'possibly tolerated' mutations as 'tolerated'. If a variant originally annotated as 'missense' affected an isoform that was missing in the newer Ensembl 95, we assumed that it was 'tolerated'.

**Immunoprecipitation**

Lysates from NL20 cells were prepared in RIPA buffer (150 mM NaCl, 1% NP-40, 0.5% sodium deoxycholate, 0.1% SDS and 50 mM Tris-HCl pH 7.5) containing protease and phosphatase inhibitors (0.2 mM PMSF, 7 mM $OV_4$ and 1x complete Mini EDTA-free Protease Inhibitor Cocktail Tablets). 5 mg of protein were immunoprecipitated overnight at 4ºC using 1 µg antibody per µg of total protein (Anti-BRG1 (G-7), sc-17796, Santa Cruz Biotechnology). In each experiment, one sample with an irrelevant antibody (anti-IgG) was included as a negative control for nonspecific binding. Immune complexes were recovered by adding 200 µL of Dynabeads Protein G (Cat#10004D) and incubating the

samples for 3 hours at 4ºC. Beads were washed three times with 1x PBS containing protease inhibitors. Final elution was performed with 8 M urea 8 M in 0.1 M Tris-HCl pH 8.

**Sample preparation for proteomic analysis**

Eluates were digested by means of the standard FASP protocol. Proteins were reduced (15 mM TCEP, 30 min, RT), alkylated (50 mM CAA, 20 min in the dark, RT) and sequentially digested with Lys-C (Wako) (protein:enzyme ratio 1:50, o/n at RT) and trypsin (Promega) (protein:enzyme ratio 1:100, 6 h at 37 °C). Resulting peptides were desalted using $C_{18}$ stage-tips.

**Mass spectrometry**

LC-MS/MS was done by coupling an UltiMate 3000 HPLC system to a Q Exactive Plus mass spectrometer (Thermo Fisher Scientific). Peptides were loaded into a trap column Acclaim™ PepMap™ 100 C18 LC Columns 5 µm, 20 mm length) for 3 min at a flow rate of 10 µl/min in 0.1% FA. Then peptides were transferred to an analytical column (PepMap RSLC C18 2 µm, 75 µm x 50 cm) and separated using a 90 min effective linear gradient (buffer A: 4% ACN, 0.1% FA; buffer B: 100% ACN, 0.1% FA) at a flow rate of 250 nL/min. The gradient used was: 0-3 min 4% B, 5-7.5 min 6% B, 7.5-60 min 17.5% B, 60-72.5 min 21.5% B, 72.5-80 min 25% B, 80-94 min 42.5% B, 94-100 min 98% B, 100-104.5 min 4% B, 105-110 min 0% B. The mass spectrometer was operated in a data-dependent mode, with an automatic switch between MS (350-1400 m/z) and MS/MS scans using a top 15 method (intensity threshold signal ≥ 3.9e4, z ≥2). MS spectra were acquired in the Orbitrap with a resolution of 70,000 FWHM (200 m/z) and MS/MS

spectra with a resolution of 17,500 FWHM (200 m/z). An active exclusion of 40 sec was used. Peptides were isolated using a 2 Th window and fragmented using higher-energy collisional dissociation (HCD) with normalized collision energy of 27. The ion target values were 3E6 for MS (25 ms max injection time) and 1E5 for MS/MS (90 ms max injection time).

**Mass spectrometry data analysis**

Raw files were processed with MaxQuant (v 1.6.2.6a) using the standard settings against a human protein database (UniProtKB/Swiss-Prot, 20,373 sequences) supplemented with contaminants. Label-free quantification was done with match between runs (match window of 0.7 min and alignment window of 20 min). Carbamidomethylation of cysteines was set as a fixed modification whereas oxidation of methionines and protein N-term acetylation as variable modifications. Minimal peptide length was set to 7 amino acids and a maximum of two tryptic missed-cleavages were allowed. Results were filtered at 0.01 FDR (peptide and protein level).

Afterwards, the "proteinGroup.txt" file was loaded in Perseus (v1.6.0.7) for further statistical analysis. Missing values were imputed from the observed normal distribution of intensities. To define potential interactors, a one-sided T-test was performed requiring at least two LFQ valid values in the "bait" group, FDR<0.15 and a $\log_2$ratio > 2.

***In silico* analysis of the SWI/SNF complex in lung adenocarcinoma patients**

We downloaded mutation, gene expression, and clinical data of LUAD patients from The Cancer Genome Atlas (TCGA-LUAD project, last updated October 1, 2019). We used the R packages 'TCGAbiolinks' (v2.12.3, R version 3.6.1) and

‘cgdsr’ (v1.3.0, R version 3.6.1). For the mutation data, we used the variant calls from the Mutect2 pipeline (N = 567 patients) and we restricted the analysis to the following mutation types: missense_variant, stop_gained, frameshift_variant, splice_acceptor_variant, splice_donor_variant, inframe_insertion, inframe_deletion, start_lost, and stop_lost.

Tumor mutation burden (TMB) estimates for the TCGA-LUAD cohort were estimated by Hoadley et al [4] and they were downloaded from https://gdc.cancer.gov/about-data/publications/PanCan-CellOfOrigin, file "mutation-load-updated.txt". The rate of non-silent mutations per Mb was used as the TMB value. Values of TMB = 0 were assumed to be errors and therefore they were excluded from the analysis.

**Real-time quantitative polymerase chain reaction**

Real-time quantitative PCR (RT-qPCR) was optimized using the Applied Biosystems 7900HT Real-Time PCR System with cDNA prepared after a reverse transcription of 1 µg total RNA (RevertAid RT Kit, Thermo Scientific). All qPCR reactions followed the KAPA SYBR® FAST qPCR Master Mix recommendations. Relative expression was calculated using *GAPDH* as housekeeping gene and applying the DDCt method. Primers for each gene are shown in ***Supplementary Table S3***. All experiments were carried out in duplicate or triplicate.

**Statistical analysis**

Unless otherwise specified, all statistical analyses were performed using R (version 3.6.1). Normality of the data was assessed using quantile-quantile plots and data transformations and statistical tests were chosen accordingly.

We performed univariate and stepwise multivariate Cox Proportional-Hazards regressions using the R package ‘survival’ (v2.44-1-1) on TCGA-LUAD mutation data of 20 SWI/SNF genes and the top 10 LUAD driver genes, as well as TMB and clinical covariates (age at diagnosis, gender and tumor stage). We considered mutations on SWI/SNF genes as one binary variable that classifies patients in those with at least one mutated SWI/SNF subunit and those with wild type SWI/SNF. Only variables significant at $p < 0.2$ were selected for the multivariate analysis. TMB, even though it was above the specified p value threshold in the univariate analysis, was also included in the multivariate regression to determine whether SWI/SNF mutational status was a prognostic factor independent of the mutational load. Kaplan-Meier curves were drawn with the R package ‘survminer’ (v0.4.6) and compared with the log-rank test.

## 2) Supplemental Note:

### Performance of different mutational analysis approaches

To evaluate the performance of our non-paired analysis approaches, first we compared the results of the two non-paired pipelines. The agreement between the two approaches was high for primary tumors (***Table S6***). We thoroughly examined the mutations uniquely found by one of the two approaches. Mutations found by Mutect2 but not by our BCFtools-based approach were usually present at very low frequencies, as low as 1-2 total mutant reads. However, 17 mutations in primary tumors had high depth and high mutant allele frequency and we manually ‘rescued’ them for our final mutation list. The ‘rescued’ mutations were mostly indels, suggesting that Mutect2 has more power than BCFtools for indel detection. On the other hand, mutations found by our BCFtools-based pipeline but not by Mutect2 were all confirmed on IGV. Most of them had been flagged as ‘germline’ by Mutect2, possibly due to their presence in gnomAD, albeit at frequencies far below 0.01. We concluded that there was insufficient evidence to exclude such mutations. Therefore, for our final analysis, we combined the full results from our BCFtools-based pipeline with the mutations we ‘rescued’ from Mutect2.

We also compared the results from our nonpaired and paired analyses in our 27 tumor-normal pairs after the ‘rescuing’ step. Out of the 65 mutations from our nonpaired analysis, 56 (86%) were also found by the paired analysis. Only 13 of the 69 (19%) paired mutations were uniquely found by the paired analysis. They were all mutations present at very low frequencies (as low as 1-2 total mutant reads) and, therefore, they were filtered by our non-paired analysis.

## 3) Supplemental References:


1. Martin M. Cutadapt removes adapter sequences from high-throughput sequencing reads. *EMBnetjournal* **2011**;17:3–12.
2. García-Alcalde F, Okonechnikov K, Carbonell J, Cruz LM, Götz S, Tarazona S, Dopazo J, Meyer TF, Conesa A. Qualimap: evaluating next-generation sequencing alignment data. *Bioinformatics* **2012***;*28:2678–2679.
3. Ewels P, Magnusson M, Lundin S, Käller M. 2016. MultiQC: summarize analysis results for multiple tools and samples in a single report. *Bioinformatics* **2016***;*32:3047–3048.
4. Hoadley KA, Yau C, Hinoue T, et al. Cell-of-Origin Patterns Dominate the Molecular Classification of 10,000 Tumors from 33 Types of Cancer. *Cell* **2018**; 173(2):291-304.

## 4) Supplemental Figure Legends:

***Supplemental Fig. S1:*** Lung SWI/SNF complex composition: Protein-protein interactions in NL20 after SMARCA4 immunoprecipitation. SWI/SNF subunits are depicted in red. Non-SWI/SNF subunits are shown in gray. Color intensity is correlated with the number of peptides found.

***Supplemental Fig. S2***: (**A**) Mutation profile of the 20 lung SWI/SNF complex subunits in the TCGA-LUAD cohort. Y axis represents all the subunits that had a genetic alteration in at least one LUAD patient. X axis contains the TCGA-LUAD patients that had at least one mutation in a lung SWI/SNF gene. On the left, mutation frequencies of these lung SWI/SNF subunits within TCGA-LUAD (total N = 567). **(B)** Functional prediction of the mutations found in SWI/SNF subunits in our 70 LUAD patients, or in TCGA-LUAD data using SIFT.

***Supplemental Fig. S3***: Downregulation of lung SWI/SNF complex subunits in our patient cohort. The $\log_2$-relative expression between each tumor and its matched normal sample was estimated as $\Delta$Ct(normal) - $\Delta$Ct(tumor). The red dots and lines represent the mean and standard deviation of the $\log_2$-relative expression values. The FDR-corrected p values from one-sample t tests under the null hypothesis that the $\log_2$-relative expression values are equal to 0 are shown.

***Supplemental Fig. S4***: (**A-J**) Kaplan-Meier curves grouping the TCGA-LUAD cohort by the mutational status of each of the top 10 LUAD driver genes.

## Supplemental Fig S1

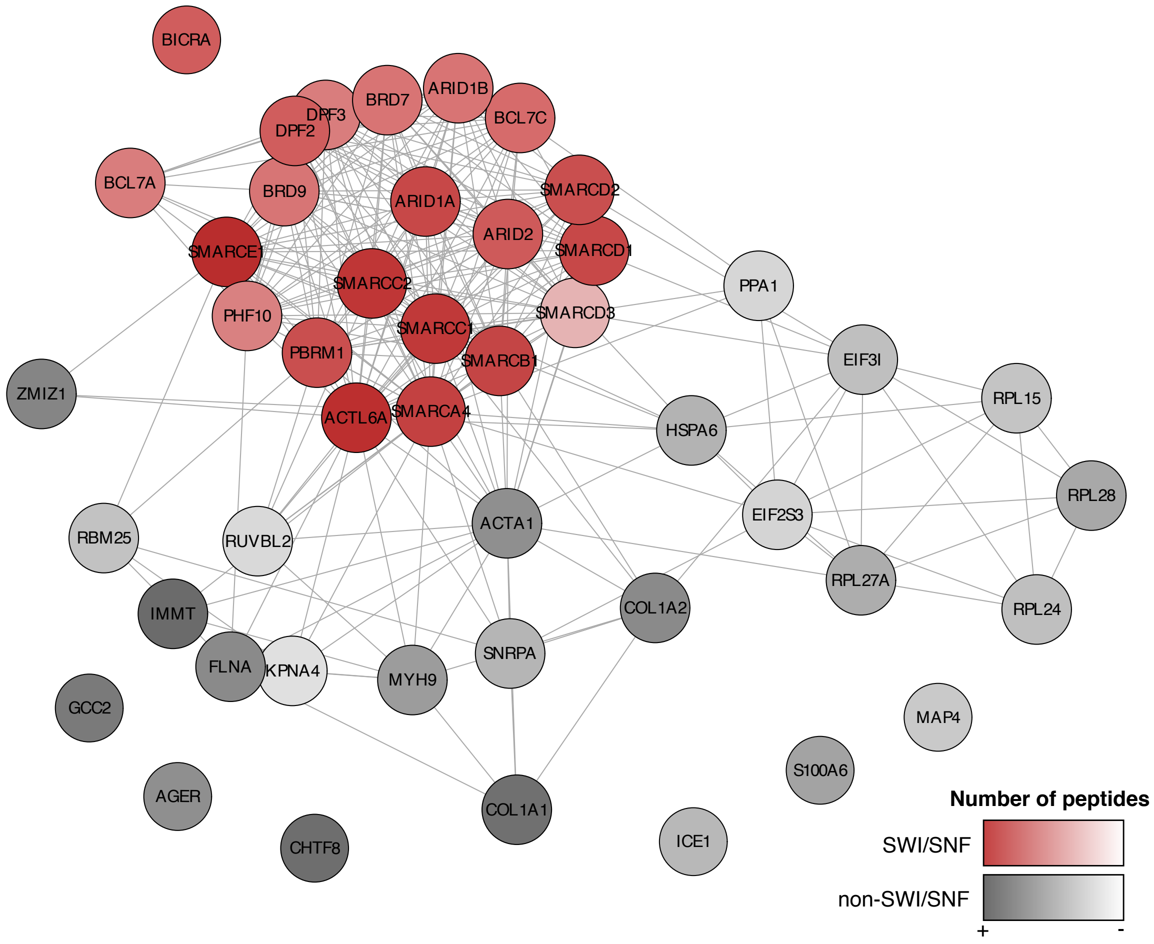

# Supplemental Fig S2

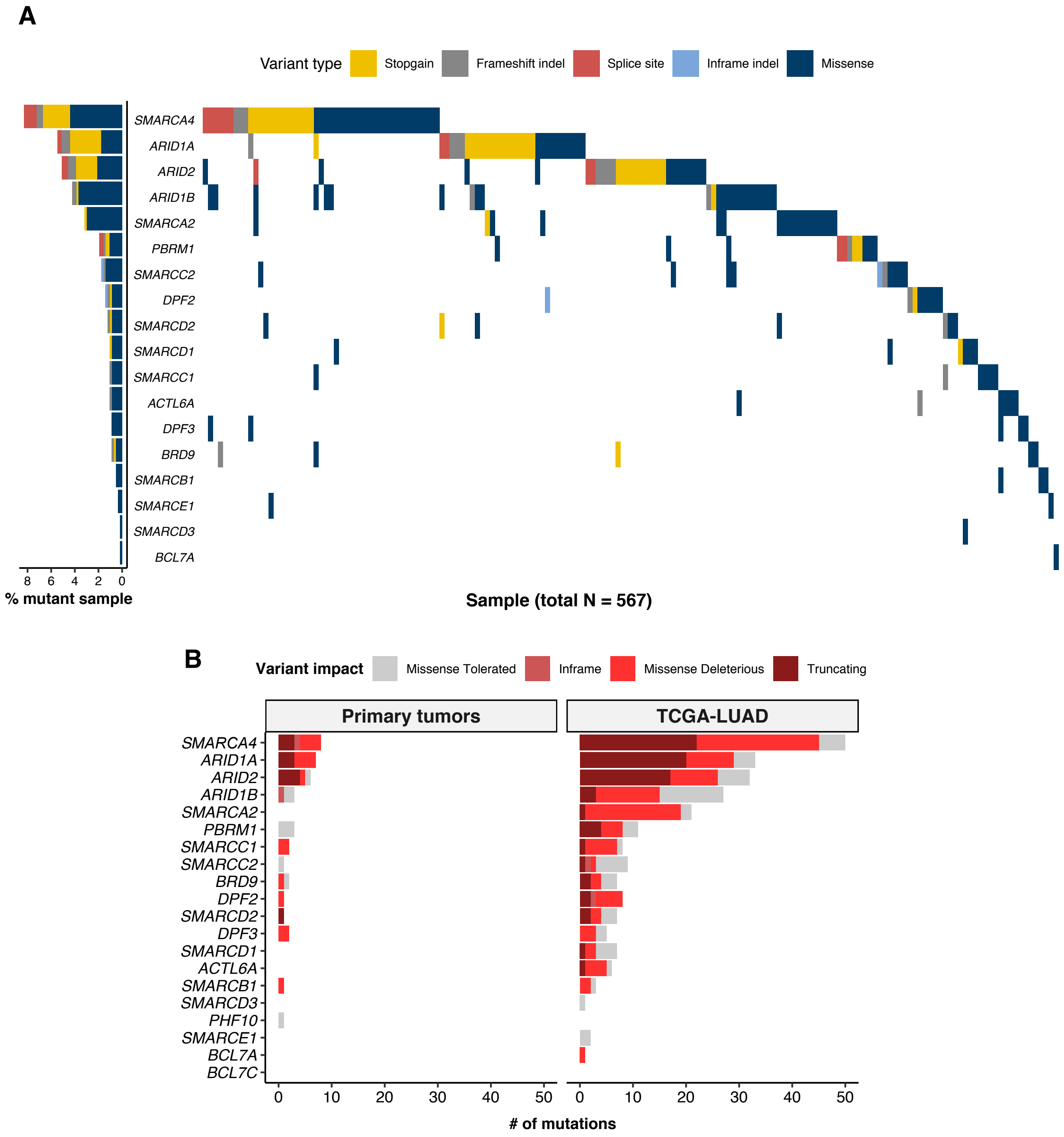

## Supplemental Fig S3

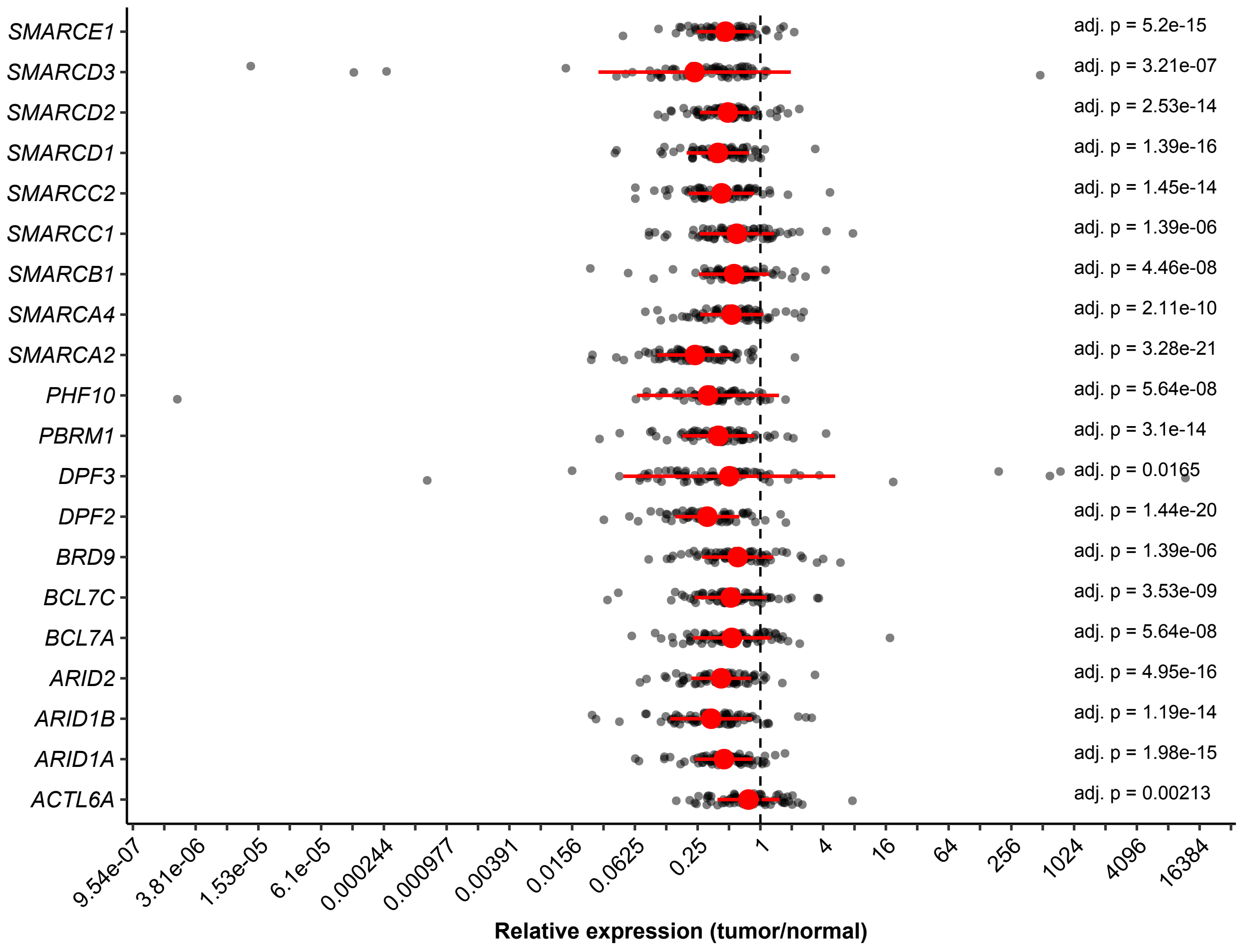

# Supplemental Fig S4

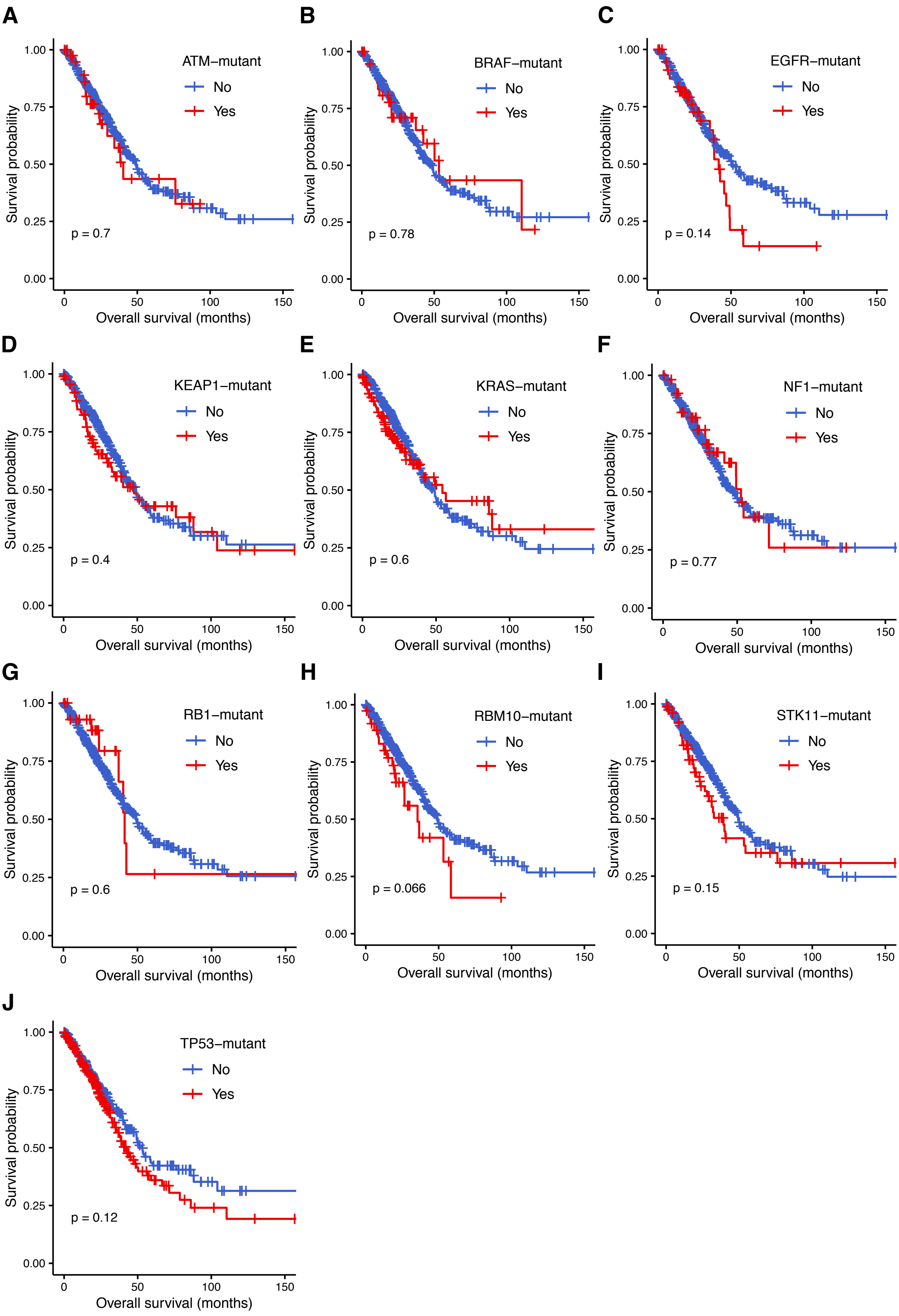

Supplement: Supplementary file 1 — Additional file 1. Supplementary Methods, Supplementary Note and Supplementary Figures S1–S4. [file 13148_2022_1261_MOESM1_ESM.pdf]
